# A non-targeted metabolite profiling pilot study suggests that tryptophan and lipid metabolisms are linked with ADHD-like behaviours in dogs

**DOI:** 10.1186/s12993-016-0112-1

**Published:** 2016-09-29

**Authors:** Jenni Puurunen, Sini Sulkama, Katriina Tiira, Cesar Araujo, Marko Lehtonen, Kati Hanhineva, Hannes Lohi

**Affiliations:** 1 Department of Veterinary Biosciences and Research Programs Unit, Molecular Neurology, University of Helsinki and Folkhälsan Research Center, Biomedicum Helsinki, P.O.Box 63, 00014 Helsinki, Finland; 2 The Folkhälsan Research Center, Helsinki, Finland; 3 School of Pharmacy, University of Eastern Finland, Kuopio, Finland; 4 Institute of Public Health and Clinical Nutrition, University of Eastern Finland, Kuopio, Finland; 5 LC–MS Metabolomics Center, Biocenter Kuopio, Kuopio, Finland

**Keywords:** Dog, ADHD, Non-targeted metabolite profiling, Metabolomics

## Abstract

**Background:**

Attention deficit hyperactivity disorder (ADHD) is a prevalent and multifactorial neuropsychiatric disorder in the human population worldwide. Complex etiology and clinical heterogeneity have challenged the research, diagnostics and treatment of the disease. Hyperactive and impulsive behaviour has also been observed in dogs, and they could offer a physiologically relevant model for human ADHD. As a part of our ongoing study to understand the molecular etiology of canine anxiety traits, this study was aimed to pilot an approach to identify metabolic biomarkers in canine ADHD-like behaviours for research, diagnostics and treatment purposes.

**Methods:**

We collected fresh plasma samples from 22 German Shepherds with varying ADHD-like behaviours. All dogs were on the same controlled diet for 2 weeks prior to sampling. A liquid chromatography combined with mass spectrometry (LC–MS)-based non-targeted metabolite profiling was performed to identify plasma metabolites correlating with the ADHD-like behaviour of the dogs.

**Results:**

649 molecular features correlated with ADHD-like behavioural scores (p_raw_ < 0.05), and three of them [sn-1 LysoPC(18:3), PC(18:3/18:2) and sn-1 LysoPE(18:2)] had significant correlations also after FDR correction (pFDR < 0.05). Phospholipids were found to negatively correlate with ADHD-like behavioural scores, whereas tryptophan metabolites 3-indolepropionic acid (IPA) and kynurenic acid (KYNA) had negative and positive correlations with ADHD-like behavioural scores, respectively.

**Conclusions:**

Our study identified associations between canine ADHD-like behaviours and metabolites that are involved in lipid and tryptophan metabolisms. The identified metabolites share similarity with earlier findings in human and rodent ADHD models. However, a larger replication study is warranted to validate the discoveries prior to further studies to understand the biological role of the identified metabolites in canine ADHD-like behaviours.

**Electronic supplementary material:**

The online version of this article (doi:10.1186/s12993-016-0112-1) contains supplementary material, which is available to authorized users.

## Background

Attention deficit hyperactivity disorder (ADHD) is a multifactorial neuropsychiatric disorder with a high prevalence (5–10 %) among children worldwide. It is also increasingly reported in adults [1–3]. In general, ADHD is defined by age-inappropriate levels of inattention, impulsivity and hyperactivity [4]. The symptoms can be disabling, interfering with normal everyday life [5]. Moreover, ADHD tends to comorbid with other neuropsychiatric disorders such as anxiety disorders [4]. Although we know that ADHD is highly heritable with mean heritability estimates of 0.7, the exact molecular mechanisms underlying ADHD pathology are still not properly understood due to the genetic complexity and interactions between genetic and environmental factors [3, 4, 6]. However, studies suggest that disruptions in normal functions of dopaminergic, serotonergic and noradrenergic systems may play a key role in ADHD pathogenesis [1, 3, 4].

One potential approach to unravel the biological pathways of ADHD is to utilise animal models, like dogs, which spontaneously show ADHD-like behaviours, such as hyperactivity, impulsivity and inattention. Many young dogs often show hyperactive and impulsive behaviour, but some hunting and working breeds, like the German Shepherd and the Belgian Shepherd, may continue to show these behavioural extremes later in life as well [7]. Due to the physiological similarities, dogs may serve as excellent natural large animal models of human ADHD. Moreover, genetic and pharmacological studies have already suggested that the underlying molecular mechanisms of ADHD behaviours may be shared in dogs and humans [8]. Dogs with high impulsivity scores have been observed to have reduced tolerance for a delay in reward and also lower levels of urinary serotonin and serotonin/dopamine ratio levels, thus, demonstrating convergent validity for canine model of impulsivity [9]. A dopamine transporter polymorphism was also found to associate with high activity levels in Belgian Malinois [10].

One of the challenges in ADHD research lies in the genetic complexity of the disorder. It is expected that multiple small effect genes contribute to ADHD susceptibility [3, 4]. There is a need either for large study cohorts or new natural models with a simpler genetic architecture such as dogs combined with complementary omics approaches. High-throughput technologies, such as metabolomics, may have the potential to facilitate ADHD research. Non-targeted metabolite profiling offers a hypothesis-free approach to detect altered metabolites and pathways in neuropsychiatric disorders. Few successful examples exist and suggest genetic and environmental contributions to diseases [11–13].

In this study, we investigated the association between plasma metabolites and ADHD-like behavioural scores in diet-controlled dogs with varying ADHD-like behaviours in order to identify ADHD-related pathways and biomarker candidates using a LC-qTOF-MS –based approach. Our results reveal associations between ADHD-like behaviours and plasma phospholipids and tryptophan metabolites in dogs.

## Methods

### Animals and study design

Data on dog ADHD-like behaviour was collected using our validated owner-completed behavioural survey [14]. The questionnaire was advertised to Finnish dog owners and breed clubs of all breeds via Facebook. Both dogs with hyperactive, impulsive and inattentive behaviours, as well as dogs with no sign of hyperactivity, impulsivity or inattention, were invited to participate in the survey.

The owner-completed behavioural survey included both general questions concerning the details of their dogs’ background, daily routines, and everyday behaviour and 13 more specific questions concerning the activity, impulsivity and inattention behaviour of the dog (Additional file 1: Table S1). In the questionnaire, we utilised the previously validated questions on impulsivity and activity levels in dogs [15, 16]. During data collection, the questionnaire was modified three times, resulting in four slightly different versions of the questionnaire (the first survey was a paper version, whereas the three others were online questionnaires). The main questions regarding our target trait, ADHD-like behaviours, were not changed between the versions. The main difference came from adding further background questions to versions three and four (maternal care, place of birth, type of food, extra nutrients, time spent alone/day, daily exercise) to better document the early life experiences and conditions of the dogs. To sort out the dogs with extreme ADHD-like behaviours, factor analysis was used to explore the factorial structure of the questionnaire, and to reveal possible interconnection between the questions concerning activity, impulsivity and inattention. The factor analysis (PROC FACTOR) was performed with SAS (version 9.3) and was conducted using the principal factor method with VARIMAX rotation. Based on the criterion of eigenvalues >1, all 13 questions were grouped into two factors, identified as ‘inattention’ and ‘impulsivity-activity’ (‘impulsivity-activity’ referred to as ‘impulsivity’ from now on). These two factors were very similar to factors found in the earlier studies [15, 16]. The ‘inattention’—factor consisted of questions #1,2,3,4,7,10 and 12, and the ‘impulsivity’—factor of questions #5,6,8,9 and 13. Question #11 did not load with either factor and was excluded. Average scores for both inattention and impulsivity factors were calculated for each dog by summing up the points in each individual question and dividing the result by the number of questions in the factor (7 for ‘inattention’ and 5 for ‘impulsivity’). The total ADHD score consisting of the mean of the answers to all the 13 questions concerning the hyperactive, impulsive and inattentive behaviour of the dog was defined for each dog to reflect the total ADHD-like status. All questions had four choices of increasing frequency (never = 1, sometimes = 2, often = 3, very often = 4). Higher scores represented higher ADHD-like behaviours.

Based on the ADHD-like behavioural scores, 22 privately-owned German Shepherds with scores varying from high to low, reflecting the ADHD-like behaviour of the dog, were recruited for the metabolomics study (Additional file 2: Table S2). Furthermore, the behaviour of the selected dogs was confirmed by owner interviews. The age of the dogs varied from 16 to 91 months, where the mean age was 62 months (median 64.6 months). The data consisted of six males (mean age 58.3 months, ranging from 16 to 86 months) and sixteen females (mean age 63.4 months, ranging from 21 to 91 months). To control the possible effects of diet on the metabolite profiles, all recruited dogs were fed with the same commercial dry food (Royal Canin Maxi Sensible) for 2 weeks prior to sampling with 1 week as a run-in period, during which, the dogs adapted to the diet change. The owners were instructed not to use any other foods or dietary supplements during the two-week period and were asked to report any changes. To investigate the metabolite profiles of the dogs, blood samples were collected from each dog by the same trained person followed by immediate isolation of plasma by a portable centrifuge. Plasma samples were kept on ice during shipping and stored in −20 degrees (max 2 months) prior to metabolomics analysis. Most of the samples were taken at the dog’s home and two samples were taken in our laboratory. Samples were collected during the day and in the evening. Most of the dogs (19 out of 22) fasted 12 h before sampling. Samples were collected with the owners’ consent under valid ethical licenses (ESAVI/6054/04.10.03/2012 and Royal Canine ethical board 30052016).

### Non-targeted LC–MS metabolite profiling analysis

The sample preparation, instrument parameters and pre-processing of raw data were performed in the LC–MS Metabolomics Center at Biocenter Kuopio (University of Eastern Finland), and they are previously presented in detail [17]. Briefly, methanol (300 µl) was used to precipitate the proteins and extract the metabolites from plasma (100 µl). The non-targeted metabolite profiling was carried out using the UHPLC-qTOF-MS system (Agilent Technologies, Waldbronn, Karlsruhe, Germany), which consisted of a 1290 LC system, a Jetstream electrospray ionization (ESI) source and a 6540 UHD accurate-mass quadrupole-time-of-flight (qTOF) mass spectrometry. All samples were analysed using two different chromatographic techniques; i.e., reversed phase (RP) and hydrophilic interaction chromatography (HILIC). In addition, data were acquired in both ionization polarities; i.e., ESI positive (ESI+) and ESI negative (ESI−).

### Non-targeted metabolomics data analysis

#### Data collection and statistical analysis

The LC–MS data was collected using the vendor’s software MassHunter Qualitative Analysis B.05.00 (Agilent Technologies), where the ions were extracted to compounds utilising the “Find by molecular feature” algorithm. The data were output as compound exchange format (.cef) files into the Mass Profiler Professional software (MPP 2.2, Agilent Technologies) for compound alignment and data pre-processing. In order to reduce noise and remove insignificant metabolite features, only the features found in at least 50 % of the samples were included in the analysis. This resulted in a dataset comprising 7058 features in four separate analytical runs [1462 in HILIC ESI(+), 1483 in HILIC ESI(−), 2624 in RP ESI(+), and 1489 in RP ESI(−)].

The four datasets were exported into Microsoft Excel (2013), and filtered according to peak area >20,000 to exclude small and insignificant features from further analysis. To investigate the associations between the peak areas of the metabolites and each of the ADHD-like behavioural scores (total, inattention and impulsivity scores), the Spearman correlation analysis was used. The results were adjusted for multiple comparisons by the Benjamini-Hochberg false discovery rate (FDR) correction [18] used in each of the four analytical approaches. Only metabolites with p_raw_ <0.05 were included in the identification analysis, and metabolites with pFDR <0.05 were considered statistically significant. Finally, the remaining features in the lists were inspected in the LC–MS chromatograms and spectra using the MassHunter software to locate chromatographic peaks with poor retention time accuracy and peak symmetry, which were removed from downstream analysis. Peak lists were also investigated to ensure that the molecular ion of a compound was included into automatic MS/MS fragmentation, and if not, targeted MS/MS analysis was performed.

To investigate whether sex, age or fasting status had any effects on the associations between the metabolites and ADHD-like behavioural scores, a partial correlation analysis including sex, age and fasting status as covariates was performed. All the statistical analyses were performed using the R project for Statistical Computing version 3.0.1.

#### Identification of the molecular features in the LC–MS data

The identification of metabolites was based on the accurate mass and isotope information; i.e., ratios, abundances and spacing, as well as product ion spectra (MS/MS) acquired either in the automatic MS/MS analysis, during the initial data acquisition, or via re-injection of the samples in targeted MS/MS mode. The spectra were compared with the METLIN Metabolite Database [19], the Human Metabolome Database (HMDB) [20], and LipidMaps [21] or fragmentation patterns reported in earlier publications. The identification of lipids was based on their characteristic fragmentation patterns reported in earlier publications [22–25]. Briefly, the key elements for identification were the protonated head group (*m/z* 184.07 for PCs and LysoPCs and *m/z* 196.03 for LysoPEs), as well as the deprotonated fatty acid fragments visible in the negative ionization mode. For LysoPCs, the product ion at *m/z* 104 was used to distinguish sn-1 and sn-2 isomers. The MS/MS fragmentation data for all of the identified metabolites is presented in Table 1.

**Table 1:** Characteristics of the putatively identified marker metabolites in liquid chromatography-mass spectrometry analysis

Column	Ionization mode	MW	*m/z*	RT (min)	Putative annotation	CID (eV)	MS/MS fragmentation	Identification reference^a^
HILIC	ESI+	231.148	232.155	1.59	Unknown metabolite, putative carnitine	10	232.1531, 85.0290, 173.0782, 95.0856, 60.0803	MS/MS
HILIC	ESI+	136.064	137.071	2.22	1-methylnicotinamide	20	94.0654, 137.0688, 65.0379, 77.0375	MID274
RP	ESI+	189.043	190.050	3.23	Kynurenic acid (KYNA)	10	190.0496, 144.0443, 172.0426	MID5683
HILIC	ESI−	165.079	164.071	3.91	Phenylalanine	10	164.0713, 103.0565, 147.0448, 90.0116, 72.0086	MID28
RP	ESI+	175.064	176.071	5.01	Indoleacetic acid	10	130.0656, 176.0780, 51.0227, 158.0587	HMDB 00197
RP	ESI+	189.079	190.086	5.77	3-Indolepropionic acid (IPA)	10	130.0655, 55.0184, 172.0754, 190.0862	MID6602
HILIC	ESI+	220.143	221.150	7.30	Unknown metabolite	10	221.1465, 84.0806, 87.0429, 90.9738, 203.1374	MS/MS
RP	ESI+	467.303	468.310	9.76	sn-1 LysoPC(14:0)	40	184.0741, 86.0970, 125.0017, 60.0814, 104.1076; ESI(−) 10 eV: 227.1995, 452.2768, 512.3016	MS/MS
RP	ESI−	563.323	562.316	9.85	sn-1 LysoPC(18:3)	10	502.2892, 277.2167, 562.3029; ESI(+) 20 eV: 184.0740, 104.1083	MS/MS
RP	ESI+	499.271	500.278	9.87	sn-1 LysoPE(20:5)	10	500.2786, 359.2548; ESI(−) 20 eV: 498.2860, 169.1368, 301.2172	MS/MS
RP	ESI+	481.319	482.326	10.03	sn-1 LysoPC(15:0)	20	184.0751, 104.1059, 482.3264; ESI(−) 10 eV: 241.2097, 466.2915	MS/MS
RP	ESI−	477.287	476.280	10.12	sn-1 LysoPE(18:2)	20	279.2321, 196.0390, 140.0058, 78.9594, 476.2834; ESI(+) 10 eV: 478.2932, 337.2703	MS/MS
RP	ESI−	479.302	478.295	10.29	sn-1 LysoPE(18:1)	10	281.2477, 478.2945, 196.0306; ESI(+) 10 eV: 480.3077, 44.0505, 62.0614, 339.2930, 462.3008	MS/MS
RP	ESI+	545.350	546.357	10.29	sn-1 LysoPC(20:3)	10	546.3626, 104.1084, 184.0740; ESI(−) 10 eV: 530.3529, 305.2466, 590.3485	MS/MS
RP	ESI+	509.350	510.357	10.42	sn-1 LysoPC(17:0)	20	184.0740, 104.1066, 510.3602; ESI(−) 10 eV: 494.3250, 269.2425, 554.3709	MS/MS
RP	ESI−	481.319	480.313	10.65	sn-1 LysoPE(18:0)	10	283.2631, 480.3112, 196.0353; ESI(+) 10 eV: 341.3049, 482.3239, 44.0494, 464.3121	MS/MS
RP	ESI−	304.241	303.235	10.70	Arachidonic acid (C20:4)	10	303.2354, 59.0219, 259.2326	LipidMaps
RP	ESI−	643.406	642.398	10.72	Unknown metabolite, putative GlcCer(d18:1/12:0) or GlcCer(d14:1/16:0)	20	642.3955, 362.1501, 363.1579, 99.9224	Hsu and Turk [25]
RP	ESI+	282.257	283.264	10.94	C18:1	10	283.2847, 43.0540, 71.0867, 149.1310, 57.0704, 101.0773	LipidMaps
RP	ESI−	647.438	646.431	10.94	Unknown metabolite, putative Cer(d18:1/24:1)	40	99.9224, 364.1699, 281.2448, 365.1777, 83.9307, 320.1746, 646.4291	Hsu and Turk [25]
RP	ESI+	584.263	585.270	11.35	Bilirubin	10	299.1388, 585.2677	MID81
RP	ESI−	847.538	846.532	11.56	PC(20:5/18:3)	40	303.2314, 102.9687, 301.2127; ESI(+) 20 eV: 184.0735, 802.5399	MS/MS
RP	ESI−	797.523	796.516	11.59	Unknown PC	10	736.4868, 796.5069; ESI(+) 20 eV: 184.0733, 752.5218	MS/MS
RP	ESI−	799.538	798.531	11.81	PC(20:4/14:0)	20	738.5067, 303.2318, 227.1970, 798.5315; ESI(+) 10 eV: 754.5405, 184.0726	MS/MS
RP	ESI−	825.555	824.547	11.83	PC(18:3/18:2)	20	764.5239, 824.5378, 277.2097, 279.2388, 45.0065; ESI(+) 20 eV: 184.0740, 780.5574, 86.0961	MS/MS
RP	ESI−	801.553	800.547	12.04	PC(18:2/16:1)	20	740.5268, 279.2323, 253.2152, 800.5403; ESI(+) 10 eV: 756.5574, 184.0733	MS/MS
RP	ESI+	743.549	744.556	12.08	PC(15:0/18:2)	10	744.5577, 184.0743; ESI(−) 20 eV: 728.5133, 788.5291, 241.2161, 279.2331	MS/MS

Metabolites with uncertain identity are also included. The characteristics include the identification references together with parameters for the LC–MS analysis, including the chromatography (column), ionization mode in the mass spectrometry (Ionization mode), *MW* molecular weight, identified ion (*m/z*), *RT* retention time, *CID* collision-induced dissociation energy, *MS/MS fragments* fragment ions in the tandem mass spectrometry

*LysoPC* lysophosphatidylcholine, *LysoPE* lysophosphatidylethanolamine, *PC* phosphatidylcholine

^a^Identification of metabolites is based on manual MS/MS spectral interpretation, METLIN ID when MS/MS spectrum available, commercial standard compound or some fragmentation patterns published earlier [25]

## Results

In this study, we used non-targeted LC–MS-based metabolite profiling to create the plasma metabolite profiles of 22 German Shepherds with varying ADHD-like behavioural scores describing the hyperactive, impulsive and inattentive behaviour of the dog. The variation in the dietary profiles was controlled by feeding the same food to all dogs for a 2-week period prior to sampling.

From the LC–MS measurements, we were able to extract 7058 molecular features to downstream statistical analysis. Of these, 649 features correlated with ADHD-like behavioural scores (p_raw_ <0.05). These molecular features were subjected to manual inspection to identify metabolites, resulting in a set of 22 identified and five unidentified metabolites (Table 1).

### Tryptophan metabolites are associated with ADHD-like behaviour

**Fig. 1:**
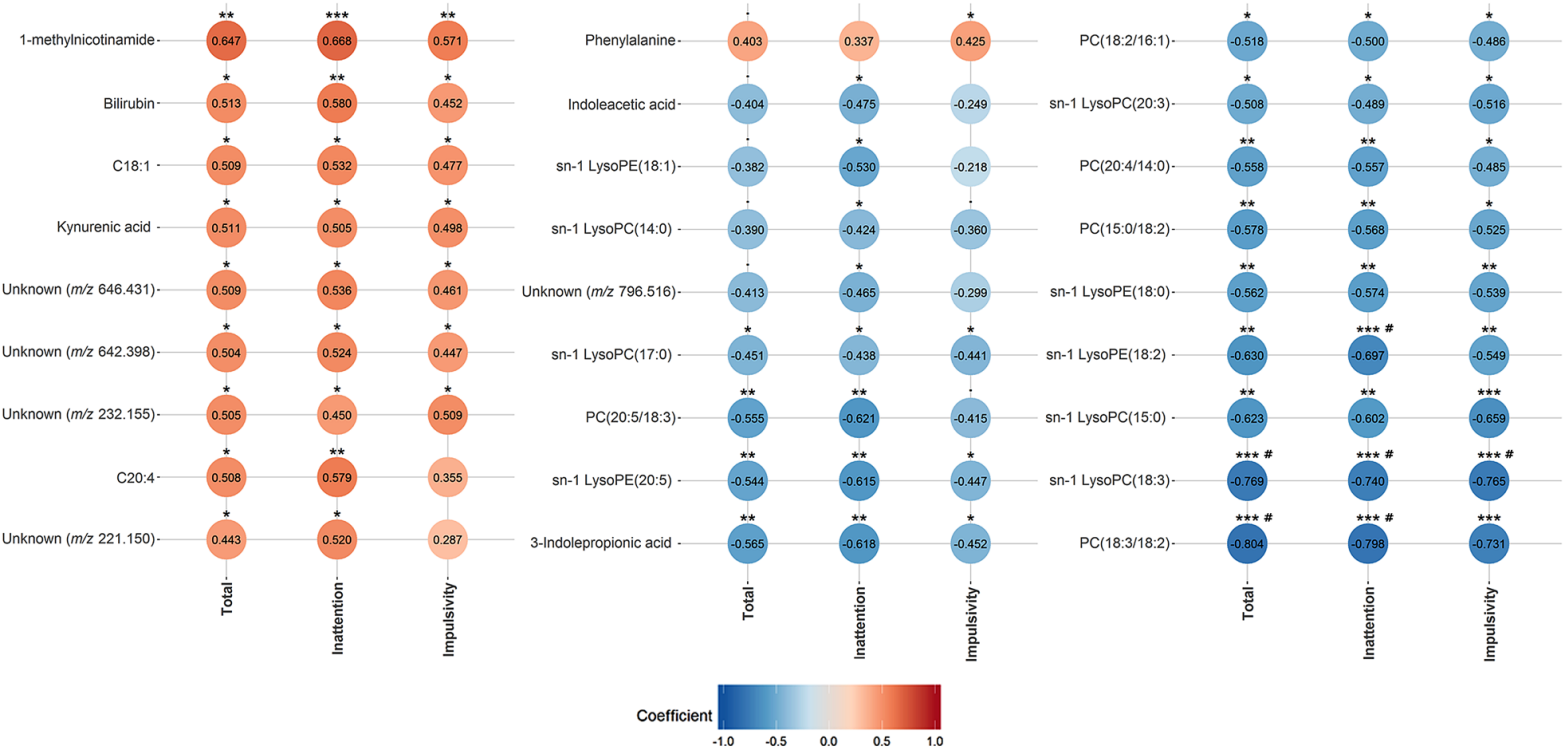
Correlation plot between metabolites and ADHD-like behavioural scores. Red and blue circles indicate positive and negative correlations, respectively. *p_raw_ < 0.05, **p_raw_ < 0.01, ***p_raw_ < 0.001, ^#^pFDR < 0.05

Three molecules related to tryptophan metabolism correlated with the ADHD-like behavioural scores (Table 2; Fig. 1). Compounds with *m/z* 190.086 and retention time (rt) 5.77 min and *m/z* 190.050 and rt 3.23 min in RP ESI(+) analysis showed identical fragmentation with 3-indolepropionic acid (IPA) (CAS No. 830-96-6) and kynurenic acid (KYNA) (CAS No. 492-27-3), respectively, according to the METLIN database (Table 1). Compound with *m/z* 176.071 and rt 5.01 in RP ESI(+) analysis was identified as indoleacetic acid (IAA) (CAS No. 87-51-4), based on its MS/MS fragmentation pattern. Higher ADHD-like behavioural scores were associated with lower plasma levels of IPA (total: r_s_ = −0.565, p_raw_ = 0.006, pFDR = 0.381; inattention: r_s_ = −0.618, p_raw_ = 0.002, pFDR = 0.241; impulsivity: r_s_ = −0.452, p_raw_ = 0.035, pFDR = 0.560) and IAA (inattention: r_s_ = −0.474, p_raw_ = 0.030, pFDR = 0.433) (Table 2; Fig. 1). In contrast, high ADHD-like behavioural scores were associated with high plasma levels of KYNA (total: r_s_ = 0.511, p_raw_ = 0.015, pFDR = 0.426; inattention: r_s_ = 0.505, p_raw_ = 0.017, pFDR = 0.392; impulsivity: r_s_ = 0.498, p_raw_ = 0.018, pFDR = 0.447).

**Table 2:** Associations between the plasma metabolites and ADHD-like behavioural scores (total ADHD, inattention, impulsivity)

Putative annotation	Total ADHD			Inattention			Impulsivity		
	r_s_	p_raw_	pFDR	r_s_	p_raw_	pFDR	r_s_	p_raw_	pFDR
Putative carnitine	0.505	0.017	0.990	0.450	0.036	0.990	0.509	0.016	0.797
1-methylnicotinamide	0.647	0.001	0.618	0.668	6.86E-04	0.455	0.571	0.005	0.461
Kynurenic acid (KYNA)	0.511	0.015	0.426	0.505	0.017	0.392	0.498	0.018	0.447
Phelynalanine	0.403	ns	ns	0.337	ns	ns	0.425	0.049	0.989
Indoleacetic acid	−0.404	ns	ns	−0.474	0.030	0.433	−0.349	ns	ns
3-Indolepropionic acid (IPA)	−0.565	0.006	0.381	−0.618	0.002	0.241	−0.452	0.035	0.560
Unknown metabolite *m/z* 221.150	0.443	0.039	0.990	0.520	0.013	0.873	0.287	ns	ns
sn-1 LysoPC(14:0)	−0.390	ns	ns	−0.424	0.049	0.510	−0.360	ns	ns
sn-1 LysoPC(18:3)	−0.769	2.93E-05	0.016	−0.740	8.26E-05	0.030	−0.765	3.32E-05	0.036
sn-1 LysoPE(20:5)	−0.544	0.009	0.409	−0.615	0.002	0.241	−0.447	0.037	0.560
sn-1 LysoPC(15:0)	−0.623	0.002	0.330	−0.602	0.003	0.241	−0.659	8.46E-04	0.320
sn-1 LysoPE(18:2)	−0.630	0.002	0.093	−0.697	3.1E-04	0.044	−0.549	0.008	0.230
sn-1 LysoPE(18:1)	−0.382	ns	ns	−0.530	0.011	0.117	−0.218	ns	ns
sn-1 LysoPC(20:3)	−0.508	0.016	0.426	−0.489	0.021	0.409	−0.516	0.014	0.437
sn-1 LysoPC(17:0)	−0.451	0.035	0.491	−0.438	0.041	0.473	−0.441	0.038	0.566
sn-1 LysoPE(18:0)	−0.562	0.006	0.146	−0.574	0.005	0.102	−0.539	0.01	0.232
Arachidonic acid (C20:4)	0.508	0.016	0.163	0.579	0.005	0.102	0.355	ns	ns
Unknown metabolite, putative GlcCer(d18:1/12:0) or GlcCer(d14:1/16:0)	0.504	0.017	0.163	0.524	0.012	0.117	0.447	0.037	0.302
C18:1	0.509	0.016	0.426	0.532	0.011	0.342	0.477	0.025	0.492
Unknown metabolite, putative Cer(d18:1/24:1)	0.509	0.016	0.163	0.536	0.010	0.117	0.461	0.031	0.283
Bilirubin	0.513	0.015	0.426	0.580	0.005	0.271	0.452	0.035	0.560
PC(20:5/18:3)	−0.555	0.009	0.150	−0.621	0.003	0.087	−0.415	ns	ns
Unknown PC *m/z* 796.516	−0.413	ns	ns	−0.465	0.029	0.173	−0.299	ns	ns
PC(20:4/14:0)	−0.558	0.007	0.148	−0.557	0.007	0.110	−0.485	0.022	0.281
PC(18:3/18:2)	−0.804	1.91E-05	0.016	−0.798	2.53E-05	0.019	−0.731	2.52E-04	0.137
PC(18:2/16:1)	−0.518	0.013	0.163	−0.500	0.018	0.137	−0.486	0.022	0.281
PC(15:0/18:2)	−0.578	0.005	0.381	−0.568	0.006	0.305	−0.525	0.012	0.416

Spearman correlation coefficients (r_s_) with statistical significance (p_raw_ and pFDR)

### More intense ADHD-like behaviour is associated with decreased plasma phospholipids, but increased fatty acids

The majority of the identified metabolites in the plasma of dogs with ADHD-like behaviours were phospholipids, including five phosphatidylcholines (PC) (PC(18:3/18:2), PC(20:5/18:3), PC(20:4/14:0), PC(18:2/16:1) and PC(15:0/18:2)), five lysophosphatidylcholines (LysoPC) (sn-1 LysoPC(18:3), sn-1 LysoPC(14:0), sn-1 LysoPC(15:0), sn-1 LysoPC(17:0) and sn-1 LysoPC(20:3)) and four lysophosphatidylethanolamines (LysoPE) (sn-1 LysoPE(18:2), sn-1 LysoPE(20:5), sn-1 LysoPE(18:1) and sn-1 LysoPE(18:0)) (Table 1). All of these detected phospholipids negatively correlated with the ADHD-like behavioural scores, of which sn-1 LysoPC(18:3), sn-1 LysoPE(18:2) and PC(18:3/18:2) had the strongest associations (Table 2; Fig. 1). The relationships between sn-1 LysoPC(18:3) and all three ADHD-like behavioural scores (total: r_s_ = −0.769, p_raw_ = 2.93E − 05, pFDR = 0.016; inattention: r_s_ = −0.740, p_raw_ = 8.26E-05, pFDR = 0.030; impulsivity: r_s_ = −0.765, p_raw_ = 3.32E-05, pFDR = 0.036), sn-1 LysoPE(18:2) and inattention score (r_s_ = −0.697, p_raw_ = 3.1E-04, pFDR = 0.044), and PC(18:3/18:2) and total and inattention scores (total: r_s_ = −0.804, p_raw_ = 1.91E-05, pFDR = 0.016; inattention: r_s_ = −0.798, p_raw_ = 2.53E-05, pFDR = 0.019) also remained significant after FDR correction. Significant correlations were not due to outliers (Additional file 3: Figure S1).

In addition, two fatty acids were identified as arachidonic acid (C20:4; *m/z* 303.235) and C18:1 *(m/z* 283.26428) (Table 1). In contrast to phospholipids, higher ADHD-like behavioural scores were associated with higher plasma levels of C20:4 (total: r_s_ = 0.508, p_raw_ = 0.016, pFDR = 0.163; inattention: r_s_ = 0.579, p_raw_ = 0.005, pFDR = 0.102) and C18:1 (total: r_s_ = 0.509, p_raw_ = 0.016, pFDR = 0.426; inattention: r_s_ = 0.532, p_raw_ = 0.011, pFDR = 0.342; impulsivity: r_s_ = 0.447, p_raw_ = 0.025, pFDR = 0.492) (Table 2; Fig. 1).

### ADHD-like behavioural scores also correlate with other plasma metabolites

The metabolite with *m/z* 585.270 and rt 11.35 in the RP ESI(+) analysis correlated positively with all three ADHD-like behavioural scores (total: r_s_ = 0.513, p_raw_ = 0.015, pFDR = 0.426; inattention: r_s_ = 0.580, p_raw_ = 0.005, pFDR = 0.271; impulsivity: r_s_ = 0.452, p_raw_ = 0.035, pFDR = 0.560) and was identified as bilirubin (CAS No. 635-65-4) (Tables 1, 2; Fig. 1). The metabolite with *m/z* 137.071 and rt 2.22 in the HILIC(+) analysis was identified as 1-methylnicotinamide (CAS No. 3106-60-3). It had a positive association with all three ADHD-like behavioural scores (total: r_s_ = 0.647, p_raw_ = 0.001, pFDR = 0.618; inattention: r_s_ = 0.668, p_raw_ = 6.86E-04, pFDR = 0.455; impulsivity: r_s_ = 0.571, p_raw_ = 0.005, pFDR = 0.461).

### The majority of the identified plasma metabolites correlate with all three ADHD-like behavioural scores

Twenty out of the 27 reported metabolites correlated with all three ADHD-like behavioural scores (total, inattention and impulsivity) (Table 2; Fig. 1). Twenty-two metabolites correlated with the total ADHD score and 26 with the inattention score. The metabolites sn-1 LysoPC(14:0), sn-1 LysoPC(18:1) and unknown PC with *m/z* 796.516 correlated only with the inattention score. The impulsivity score correlated with 21 metabolites, and phenylalanine was found to specifically correlate only with the impulsivity score.

### Age, sex and fasting have minor effects on the association between metabolites and ADHD-like behavioural scores

Since there were differences in the fasting status of the dogs (Additional file 2: Table S2), we wanted to determine whether this had any effects on the observed correlations between the plasma metabolites and ADHD-like behavioural scores. Also, the possible effects of age and sex were analysed. Correlation coefficients and p-values adjusted for age, sex and fasting are represented in Table 3 together with the original p-values. Most of the associations between the metabolites and ADHD-like behavioural scores remained after controlling for age, sex and fasting status (age, sex and fasting adjusted *p* value < 0.05). However, age, sex and fasting had significant effects on associations between IPA and the impulsivity score (original p = 0.035, adjusted p = 0.058), sn-1 LysoPC(14:0) and the inattention score (original p = 0.049, adjusted p = 0.102), sn-1 LysoPC(17:0) and the inattention score (original p = 0.041, adjusted p = 0.055), unknown PC with *m/z* 796.516 and the inattention score (original p = 0.029, adjusted p = 0.087), IAA and the inattention score (original p = 0.03, adjusted p = 0.062), phenylalanine and the impulsivity score (original p = 0.049, adjusted p = 0.063), and unknown metabolite with *m/z* 221.150 and the total ADHD score (original p = 0.039, adjusted p = 0.069).

**Table 3:** Age, sex and fasting-adjusted associations between the plasma metabolites and ADHD-like behavioural scores

Putative annotation	Total ADHD			Inattention			Impulsivity		
	Original	Adjusted		Original	Adjusted		Original	Adjusted	
	p	r_s_	p	p	r_s_	p	p	r_s_	p
Putative carnitine	0.017	0.560	0.013	0.036	0.527	0.02	0.016	0.526	0.021
1-methylnicotinamide	0.001	0.642	0.003	6.86E-04	0.671	0.002	0.005	0.576	0.010
Kynurenic acid (KYNA)	0.015	0.519	0.023	0.017	0.486	0.035	0.018	0.543	0.016
Phelynalanine	ns	ns	ns	ns	ns	ns	0.049	0.435	0.063
Indoleacetic acid	ns	ns	ns	0.030	−0.448	0.062	ns	ns	ns
3-Indolepropionic acid (IPA)	0.006	−0.578	0.010	0.002	−0.655	0.002	0.035	−0.442	0.058
Unknown metabolite *m/z* 221.150	0.039	0.426	0.069	0.013	0.475	0.040	ns	ns	ns
sn-1 LysoPC(14:0)	ns	ns	ns	0.049	−0.387	0.102	ns	ns	ns
sn-1 LysoPC(18:3)	2.93E-05	−0.782	7.54E-05	8.26E-05	−0.765	1.34E-04	3.32E-05	−0.785	6.86E-05
sn-1 LysoPE(20:5)	0.009	−0.569	0.011	0.002	−0.615	0.005	0.037	−0.499	0.030
sn-1 LysoPC(15:0)	0.002	−0.640	0.003	0.003	−0.598	0.007	8.46E-04	−0.701	8.0E-04
sn-1 LysoPE(18:2)	0.002	−0.670	0.002	0.003	−0.703	8.0E-04	0.008	−0.645	0.003
sn-1 LysoPE(18:1)	ns	ns	ns	0.011	−0.493	0.032	ns	ns	ns
sn-1 LysoPC(20:3)	0.016	−0.550	0.015	0.021	−0.555	0.014	0.014	−0.561	0.013
sn-1 LysoPC(17:0)	0.035	−0.463	0.046	0.041	−0.447	0.055	0.038	−0.499	0.030
sn-1 LysoPE(18:0)	0.006	−0.610	0.006	0.005	−0.622	0.004	0.01	−0.600	0.007
Arachidonic acid (C20:4)	0.016	0.502	0.029	0.005	0.555	0.014	ns	ns	ns
Unknown metabolite, putative GlcCer(d18:1/12:0) or GlcCer(d14:1/16:0)	0.017	0.514	0.024	0.012	0.547	0.015	0.037	0.473	0.041
C18:1	0.016	0.508	0.026	0.011	0.524	0.021	0.025	0.505	0.028
Unknown metabolite, putative Cer(d18:1/24:1)	0.016	0.506	0.027	0.010	0.529	0.020	0.031	0.488	0.034
Bilirubin	0.015	0.511	0.025	0.005	0.544	0.016	0.035	0.515	0.024
PC(20:5/18:3)	0.009	−0.601	0.008	0.003	−0.615	0.007	ns	ns	ns
Unknown PC *m/z* 796.516	ns	ns	ns	0.029	−0.404	0.087	ns	ns	ns
PC(20:4/14:0)	0.007	−0.559	0.013	0.007	−0.538	0.017	0.022	−0.531	0.019
PC(18:3/18:2)	1.19E-05	−0.804	9.91E-05	2.53E-05	−0.785	1.89E-04	2.52E-04	−0.767	3.24E-04
PC(18:2/16:1)	0.013	−0.529	0.020	0.018	−0.537	0.018	0.022	−0.476	0.039
PC(15:0/18:2)	0.005	−0.604	0.006	0.006	−0.578	0.010	0.012	−0.590	0.008

Spearman correlation coefficients (corrected r_s_) from partial correlation analysis including age, sex and fasting status as covariates together with statistical significance (adjusted p). Original p values are also shown (original p)

## Discussion

ADHD is a prevalent and severe neuropsychiatric disorder, but yet poorly characterised for underlying genes and molecular networks. Genetic complexity and clinical heterogeneity have challenged the research, warranting new approaches to identify novel biomarkers and pathways. An alternative approach would be a study of a physiologically relevant large animal model with natural ADHD-like behaviours such as the dog [15]. Here, we applied a methodologically well-controlled pilot non-targeted metabolite profiling of canine ADHD-like behaviours in order to investigate the correlation between the plasma metabolite profiles and ADHD-like behavioural scores of German Shepherds with varying ADHD-like behaviours. We report 27 metabolites that correlated with at least one of the three ADHD-like behavioural scores (total, inattention, impulsivity). The identified ADHD-like behaviour-related candidate metabolites indicate alterations in tryptophan and phospholipids metabolisms. The same pathways have been suggested in human ADHD [26–30], and the possible important overlap in the human and canine pathways warrants a larger replication study in dogs prior to further conclusions of the metabolic similarity of the ADHD models.

**Fig. 2:**
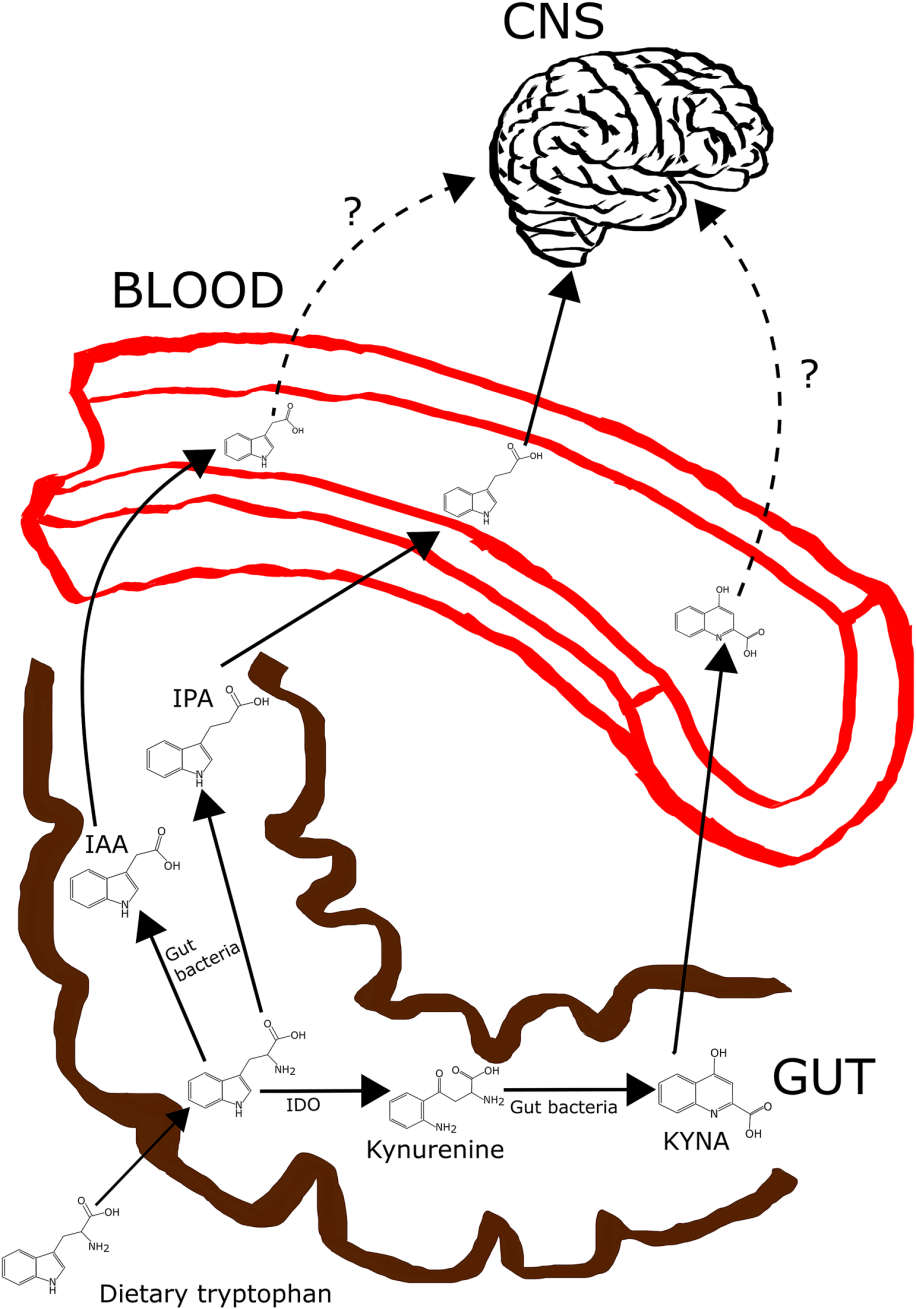
Simplified illustration of the possible metabolic pathways of dietary tryptophan in the intestines. Dietary tryptophan can be degraded in the intestines by enteric bacteria to produce IAA and IPA or KYNA via kynurenine. From the intestines, IPA, IAA and KYNA are transferred to circulation. IPA is known to cross BBB, and thus, can migrate to CNS and act there, but the ability of IAA and KYNA to cross BBB is uncertain. In addition to the degradation of dietary tryptophan in the intestines, KYNA and IAA can also be synthetised from tryptophan in various other tissues in the body, but IPA is solely produced in the intestines by enteric bacteria. *BBB* blood–brain barrier, *CNS* central nervous system, *IAA* indoleacetic acid, *IDO* indoleamine 2,3-dioxygenase, *IPA* 3-indolepropionic acid, *KYNA* kynurenic acid

We found three interesting metabolites in the tryptophan pathway, IPA, IAA and KYNA. Lower plasma IPA and IAA levels were associated with higher ADHD-like behavioural scores. IPA is a microbial deamination product of dietary tryptophan with antioxidant capacity [31, 32], produced solely by enteric bacteria (including *Clostridium sporogenes*) in the intestines [33–35] (Fig. 2). IAA is a plant hormone of the auxin class synthesised via multiple pathways in the plants [36], but it can also be produced in mammals from tryptophan in tissues, and in the intestines by enteric bacteria (including *Clostridium bartlettii*) [37, 38]. Due to the intestinal bacterial production of both IPA and IAA by bacteria belonging to the same genus (*Clostridium*), negative correlations between the ADHD-like behavioural scores and these metabolites suggest differences in intestinal microbiota of dogs with different ADHD-like behaviours, since there were no differences in the diets (and intake of tryptophan) of the dogs. Gut microbiota may have a bidirectional effect on the nervous and the immune systems [39–41]. Since IPA is capable of crossing the blood–brain barrier (BBB) and is known to have neuroprotective actions in the central nervous system (CNS) [35], decreased IPA may predispose dogs to neurological and behavioural abnormalities due to oxidative stress, for example. Alternatively, stress caused by hyperactive and impulsive behaviour may lead to altered gut microbiota and result in changed plasma IPA. Changes in the gut microbiota have been suggested in neuro behavioural disorders such as mood disorders and autism [40].

The third metabolite in the tryptophan pathway, KYNA, was positively correlated with the ADHD-like behavioural scores. KYNA is a neuroactive metabolite produced in the kynurenine pathway of tryptophan metabolism [42, 43]. It acts as an endogenous antagonist of the cholinergic nicotinic α7-receptor (α7nAChR) and the glutamatergic *N*-methyl-d-aspartate receptor (NMDAR) in the CNS and as an agonist of a particular G-protein coupled receptor, GPR35, in immune cells and in the gastrointestinal (GI) tract [44]. Interestingly, altered levels of KYNA have been proposed in schizophrenia [45, 46], depression [47] and bipolar disorder [48]. A recent study demonstrated a reduction in serum KYNA levels in adult ADHD patients, when compared with controls [26]. Interestingly, our data showed a positive relationship between the plasma KYNA levels and ADHD-like behavioural scores in adult dogs, indicating that dogs with more intense ADHD-like behaviour had higher KYNA levels. KYNA is mainly produced via tryptophan catabolism in various tissues, but enteric bacteria in the intestines are also capable of converting dietary tryptophan into KYNA [49, 50] (Fig. 2). Higher plasma KYNA levels may be due to increased bacterial production of KYNA in the intestines or increased catabolism of tryptophan via the kynurenine pathway in the tissues resulting, for example, from inflammation or oxidative stress [51]. Thus, there may be less tryptophan available for transport into the brain for serotonin synthesis. Serotonin, in turn, is an important neurotransmitter, regulating impulsivity and social behaviour [52]. Decreased serotonin levels have been reported in ADHD patients [52]. Thus, the positive correlation between the plasma KYNA levels and ADHD-like behavioural scores may indirectly reflect decreased serotonin levels with characteristic behavioural symptoms in dogs with higher ADHD-like behavioural scores.

We observed strong and negative correlations between ADHD-like behavioural scores and fifteen phospholipids, including five PCs, five LysoPCs, four LysoPEs and one unknown PC. The blood lipid composition is affected by nutrition and fasting [53], and we, therefore, changed the diet of all study dogs and controlled fasting in the statistical analysis. Thus, the observed difference suggests alterations in the endogenous phospholipid metabolism or in the absorption of dietary lipids between the study groups. Phospholipids are important signalling molecules and major components of cell membranes regulating membrane fluidity, charge and receptor function [54, 55]. The adequate amount of brain phospholipids and their right fatty acid composition is especially important to ensure optimal membrane functionality in the brain [55]. Regarding this, the lower plasma phospholipids may have negative effects on the physiology and behaviour of dogs with more intense ADHD-like behaviour.

Phospholipids are composed of hydrophobic and hydrophilic parts, which are joined together by glycerol [56]. The hydrophilic part consists of a phosphate group combined with a characteristic head group (choline in PCs and ethanolamine in PEs), whereas the hydrophobic part consists of different kinds of combinations of fatty acids, PCs containing two fatty acids, whereas LysoPCs and LysoPEs contain only one fatty acid [56]. Thus, the composition of phospholipids depends on the availability of free fatty acids, which are incorporated into phospholipids. Interestingly, both human [27, 28, 30, 57] and rodent [58–61] studies have suggested that the omega-3 polyunsaturated fatty acid (PUFA) status may contribute to the etiology of ADHD. Both children and adult ADHD patients have been demonstrated to have decreased proportions of long chain PUFAs (LC-PUFAs) and especially omega-3 PUFAs, like eicosapentaenoic acid (EPA; 20:5 n-3) and docosahexaenoic acid (DHA; 22:6 n-3), in plasma lipids when compared with control subjects [28, 29, 62–65].

It has been suggested that the relationship between decreased omega-3 PUFAs and ADHD would lie in dopamine neurotransmission, as rodent studies have proposed that chronic omega-3 PUFA deficiency is associated with significantly decreased concentrations of endogenous dopamine and reduced dopamine-2 (D_2_) receptor binding in the frontal cortex [59, 61], accompanied by attentional and behavioural problems when compared to rodents fed with diet containing adequate levels of PUFAs [66]. In this study, ADHD-like behavioural scores negatively correlated with plasma phospholipids containing combinations of omega-3 (18:3 n-3 (ALA), 20:5 n-3 (EPA)) and omega-6 (18:2 n-6 (LA)) fatty acids. Our results are not directly comparable to those findings made in ADHD patients, but indicate that phospholipid abnormalities could contribute to canine ADHD-like behaviours too. In contrast to phospholipids, ADHD-behavioural scores were positively associated with plasma levels of free fatty acids C20:4 and C18:1. This may refer to an increased breakdown of phospholipids or problems in the phospholipid synthesis, rather than problems in the intestinal absorption of dietary fatty acids.

Our study also suggests the involvement of oxidative stress in canine ADHD-like behaviours. The ADHD-like behavioural scores showed a positive relationship with fatty acid C20:4, an oxidative stress marker, but a negative relationship with the antioxidant IPA. Impaired balance between the oxidant and antioxidant systems and increased levels of oxidative stress have already been demonstrated in ADHD, although precise mechanisms remain to be characterised [67–70].

We have previously demonstrated the promise of the metabolomics approach in canine fear research [17], and now, we have applied this approach to study canine ADHD-like behaviours with better optimised methodology and preparation of the study cohort. Instead of whole blood, we used fresh plasma samples from dogs that had undergone a controlled diet change before the sampling. However, we have several limitations in our study. First, our sample size is small for conclusive results. Second, we must acknowledge that the behavioural survey is based on the owner reports of the behaviours and activity levels of the dogs, and no behavioural testing was performed to verify the owner reports. Thirdly, fasting control was incomplete, as some dogs failed it. Finally, the time of sampling varied from daytime to evening and should be less variable in the future to better control possible variations due to the circadian rhythm.

## Conclusions

Our metabolomics study suggests associations between canine ADHD-like behaviours and tryptophan and phospholipid metabolisms. A replication study with a larger sample size is needed to validate our findings and to confirm the overlap in the affected pathways between human ADHD and canine ADHD-like behaviours.

## Additional files

10.1186/s12993-016-0112-1 List of 13 questions concerning the ADHD-like behavior of the dog.

10.1186/s12993-016-0112-1 Demographics of the study participants.

10.1186/s12993-016-0112-1 Sample distributions for each metabolite having significant correlation coefficients (pFDR < 0.05).

## Abbreviations

ADHD: attention deficit hyperactivity disorder
BBB: blood-brain barrier
CNS: central nervous system
ESI: electrospray ionization
HILIC: hydrophilic interaction
IAA: indoleacetic acid
IPA: 3-indolepropionic acid
KYNA: kynurenic acid
LC: liquid chromatography
LysoPC: lysophosphatidylcholine
LysoPE: lysophosphatidylethanolamine
MS: mass spectrometry
PC: phosphatidylcholine
RP: reversed phase
