# Supplementary material for: A non-targeted metabolite profiling pilot study suggests that tryptophan and lipid metabolisms are linked with ADHD-like behaviours in dogs
Source: Behav Brain Funct. 2016 Sep 29;12:27. doi: 10.1186/s12993-016-0112-1 (PMC5043524; doi:10.1186/s12993-016-0112-1)
Supplement: Supplementary file 1 — 10.1186/s12993-016-0112-1 List of 13 questions concerning the ADHD-like behavior of the dog. [file 12993_2016_112_MOESM1_ESM.docx]

**Additional file 1:Table S1.**

| 1. My dog has a difficult time learning, because it is careless or other things can easily attract its attention.  2. It's easy to attract its attention, but it loses its interest soon.  3. It's difficult for it to concentrate on a task or play.  4. It leaves from its place when it should stay.  5. It cannot be quiet, it cannot be easily calmed.  6. It fidgets all the time.  7. It seems that it doesn't listen even if it knows that someone is speaking to it.  8. It is excessive, difficult to control, and if it lunges it is hard to hold back.  9. It would always play and run.  10. It solves simple tasks easily, but it often has difficulties with complicated tasks, even if it knows them and has practiced them often.  11. It is likely to react hastily and that's why it is failing tasks.  12. Its attention can be easily distracted.  13. It cannot wait as it has no self-control. |
| --- |

The questions were included in the owner-completed behavioral questionnaire (Vas et al., 2007)**.** The scale: Never=1 Sometimes=2 Often=3 Very often=4.
