# Supplementary material for: A non-targeted metabolite profiling pilot study suggests that tryptophan and lipid metabolisms are linked with ADHD-like behaviours in dogs
Source: Behav Brain Funct. 2016 Sep 29;12:27. doi: 10.1186/s12993-016-0112-1 (PMC5043524; doi:10.1186/s12993-016-0112-1)
Supplement: Supplementary file 2 — 10.1186/s12993-016-0112-1 Demographics of the study participants. [file 12993_2016_112_MOESM2_ESM.docx]

**Additional file2: Table S2.**

|  | **Age (months)** | **Sex** | **Total ADHD score** | **Inattention score** | **Impulsivity-activity score** | **Fasting prior to blood sampling** |
| --- | --- | --- | --- | --- | --- | --- |
|  |  |  |  |  |  |  |
| **1** | 57 | female | 3.5 | 3.1 | 3.8 | yes |
| **2** | 70 | female | 3.2 | 3.1 | 3.4 | yes |
| **3** | 91 | female | 3.1 | 2.6 | 3.8 | yes |
| **4** | 63 | female | 3.1 | 3.0 | 3.0 | yes |
| **5** | 67 | female | 3.1 | 3.1 | 3.0 | yes |
| **6** | 73 | male | 2.8 | 2.3 | 3.8 | yes |
| **7** | 75 | male | 2.5 | 2.6 | 2.4 | yes |
| **8** | 16 | male | 2.3 | 1.9 | 3.0 | yes |
| **9** | 25 | female | 1.9 | 2.3 | 1.6 | yes |
| **10** | 65 | female | 1.9 | 2.0 | 2.0 | yes |
| **11** | 65 | female | 1.8 | 1.4 | 2.4 | no |
| **12** | 86 | male | 1.8 | 1.9 | 1.8 | yes |
| **13** | 65 | female | 1.7 | 1.6 | 1.8 | yes |
| **14** | 59 | female | 1.5 | 1.1 | 2.0 | no |
| **15** | 89 | female | 1.4 | 1.1 | 1.8 | no |
| **16** | 78 | female | 1.3 | 1.1 | 1.4 | yes |
| **17** | 21 | female | 1.2 | 1.1 | 1.4 | yes |
| **18** | 35 | male | 1.2 | 1.3 | 1.0 | yes |
| **19** | 65 | male | 1.1 | 1.1 | 1.0 | yes |
| **20** | 65 | female | 1.0 | 1.0 | 1.0 | yes |
| **21** | 71 | female | 1.0 | 1.0 | 1.0 | yes |
| **22** | 67 | female | 1.0 | 1.0 | 1.0 | yes |

Detailed information including age, sex, ADHD-like behavioral scores and fasting status for each individual dog is presented.
