# Supplementary material for: A non-targeted metabolite profiling pilot study suggests that tryptophan and lipid metabolisms are linked with ADHD-like behaviours in dogs
Source: Behav Brain Funct. 2016 Sep 29;12:27. doi: 10.1186/s12993-016-0112-1 (PMC5043524; doi:10.1186/s12993-016-0112-1)
Supplement: Supplementary file 3 — 10.1186/s12993-016-0112-1 Sample distributions for each metabolite having significant correlation coefficients (pFDR < 0.05). [file 12993_2016_112_MOESM3_ESM.docx]

**Additional file 3:Figure S1.**


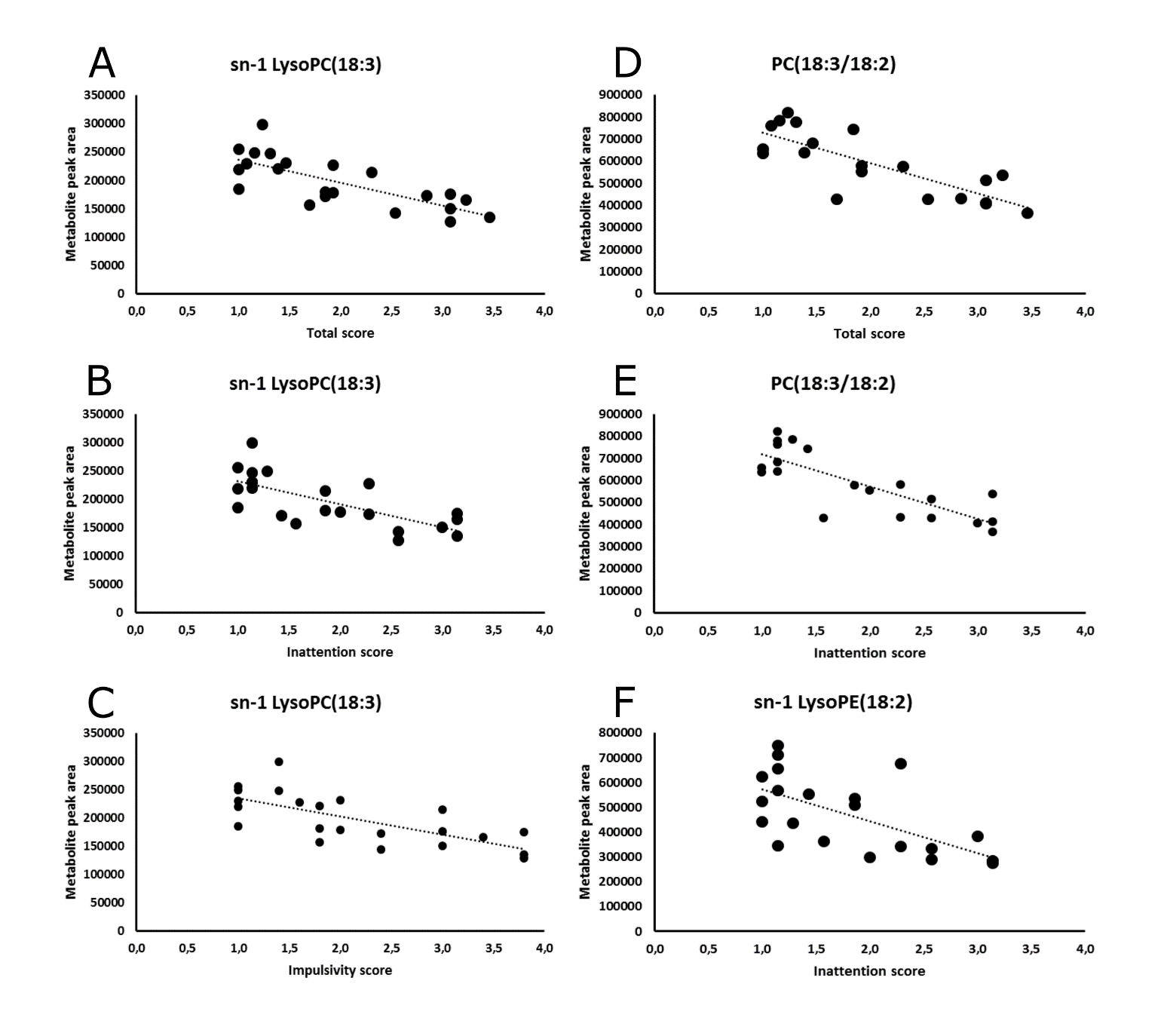
Sample distributions for sn-1 LysoPC(18:3) (A-C), PC(18:3/18:2) (D-E), and sn-1 LysoPE(18:2) (F). The ADHD-like behavioral scores (total, inattention and impulsivity) are along the x-axis, whereas the metabolite peak areas along the y-axis.
